# Year-round trace gas measurements in the central Arctic during the MOSAiC expedition

**DOI:** 10.1038/s41597-022-01769-6

**Published:** 2022-11-25

**Authors:** Hélène Angot, Byron Blomquist, Dean Howard, Stephen Archer, Ludovic Bariteau, Ivo Beck, Matthew Boyer, Molly Crotwell, Detlev Helmig, Jacques Hueber, Hans-Werner Jacobi, Tuija Jokinen, Markku Kulmala, Xin Lan, Tiia Laurila, Monica Madronich, Donald Neff, Tuukka Petäjä, Kevin Posman, Lauriane Quéléver, Matthew D. Shupe, Isaac Vimont, Julia Schmale

**Affiliations:** 1 Extreme Environments Research Laboratory, École Polytechnique Fédérale de Lausanne (EPFL) Valais Wallis, Sion, Switzerland; 2 Institute of Arctic and Alpine Research, University of Colorado, Boulder, Colorado USA; 3 Cooperative Institute for Research in Environmental Sciences, University of Colorado, Boulder, Colorado USA; 4 NOAA, Physical Sciences Laboratory, Boulder, Colorado USA; 5 Bigelow Laboratory for Ocean Sciences, East Boothbay, Maine, USA; 6 Institute for Atmospheric and Earth System Research/INAR-Physics, Faculty of Science, University of Helsinki, Helsinki, Finland; 7 NOAA, Global Monitoring Laboratory, Boulder, CO USA; 8 Institute for Geosciences and Environmental Research, Univ. Grenoble Alpes/CNRS/Grenoble-INP/IRD, Grenoble, France; 9 Climate & Atmosphere Research Centre (CARE-C), The Cyprus Institute, Nicosia, Cyprus; 10 Present Address: Boulder AIR, Boulder, Colorado USA; 11 Present Address: JH Atmospheric Instrument Design, Boulder, Colorado USA

**Keywords:** Atmospheric chemistry, Atmospheric chemistry

## Abstract

Despite the key role of the Arctic in the global Earth system, year-round *in-situ* atmospheric composition observations within the Arctic are sparse and mostly rely on measurements at ground-based coastal stations. Measurements of a suite of *in-situ* trace gases were performed in the central Arctic during the Multidisciplinary drifting Observatory for the Study of Arctic Climate (MOSAiC) expedition. These observations give a comprehensive picture of year-round near-surface atmospheric abundances of key greenhouse and trace gases, i.e., carbon dioxide, methane, nitrous oxide, ozone, carbon monoxide, dimethylsulfide, sulfur dioxide, elemental mercury, and selected volatile organic compounds (VOCs). Redundancy in certain measurements supported continuity and permitted cross-evaluation and validation of the data. This paper gives an overview of the trace gas measurements conducted during MOSAiC and highlights the high quality of the monitoring activities. In addition, in the case of redundant measurements, merged datasets are provided and recommended for further use by the scientific community.

## Abstract (summary)

**Table:** 

Measurement(s)	atmospheric ozone • atmospheric carbon dioxide • atmospheric methane • atmospheric carbon monoxide • atmospheric nitrous oxide • atmospheric dimethylsulfide • atmospheric sulfur dioxide • atmospheric gaseous elemental mercury • volatile organic compounds
Technology Type(s)	ozone analyzer • carbon dioxide analyzer • methane analyzer • carbon monoxide analyzer • nitrous oxide analyzer • dimethylsulfide analyzer • sulfur dioxide analyzer • gaseous elemental mercury analyzer • gas chromatography-mass spectrometry
Sample Characteristic - Environment	Atmosphere
Sample Characteristic - Location	central Arctic

## Background & Summary

The Arctic has warmed three times more rapidly than the rest of the planet, and this warming is happening faster than predicted^1^. The effects of climate change are thus more pronounced in the Arctic than in other climate zones, leading to e.g., large temperature increase, sea ice decline^2,3^, loss of permafrost^4^, and changes in Arctic ecology^5,6^. In addition, discovery of new petroleum and mineral resources, the opening of shipping routes in the Arctic Ocean, and geopolitical interests are posing ever-increasing pressure on the Arctic and further environmental impacts are becoming evident^7–10^. These profound regional changes might have significant impacts on mid-latitude climate variability^11,12^, highlighting the central role of the Arctic in the global Earth system.

In its Special Report on Global Warming of 1.5 °C, the Intergovernmental Panel on Climate Change (IPCC) identified human activities and associated greenhouse gas (GHG) emissions as the root cause of global warming^13^. The direct impact of short-lived climate forcers (e.g., methane (CH_4_), ozone (O_3_)) persists from a few days to a decade at most. However, due to long atmospheric lifetimes, emissions of GHGs such as carbon dioxide (CO_2_) and nitrous oxide (N_2_O) have long-lasting impacts (centuries) on radiative forcing^14^. Therefore, long-term observations of GHG atmospheric abundances are essential to evaluate the effectiveness of mitigation policies and to identify potential climate feedback processes^15^.

Monitoring GHG atmospheric abundances in the Arctic is, however, challenging because it is a remote and harsh environment with a sparsity of locations with appropriate infrastructure. As a consequence, observations have mostly been performed at ground-based coastal stations or during short-term aircraft or ship-based campaigns^16^. In that context, the Multidisciplinary drifting Observatory for the Study of Arctic Climate (MOSAiC; https://mosaic-expedition.org/) expedition offered an unprecedented opportunity to monitor the year-round atmospheric composition of the central Arctic. The backbone of MOSAiC was the year-round operation of the Research Vessel *Polarstern* which drifted with the sea ice across the central Arctic from October 2019 to September 2020. *In-situ* observations addressing key aspects of the coupled Arctic climate system were set up on-board *Polarstern* and on the surrounding sea ice. A general overview of the expedition and a description of observations carried out by the “Atmosphere” science team and the drift track can be found in Shupe *et al*.^17^.

In addition to monitoring GHGs, the expedition provided a unique platform to study the wider Arctic atmospheric chemical composition. The latest Arctic Monitoring & Assessment Programme (AMAP) report on the impacts of short-lived climate forcers on Arctic Climate^18^ highlighted the climate-relevance of other compounds such as sulfur dioxide (SO_2_; precursor of sulfate aerosols). In the period 1990–2015, the Arctic warming attributed to declining SO_2_ emissions was of similar magnitude to the warming driven by increasing CO_2_ emissions (~0.29 °C per decade). The year-long expedition also provided a platform to investigate seasonal variations. During winter and spring, the combination of increased long-range transport from mid-latitudes and of relatively weak removal processes leads to the build-up of air pollution, the so-called Arctic haze^19–22^. Previous studies have also shown that the Arctic atmosphere features a number of complex chemical and physical processes at the onset of spring^23–25^. These transformations can, for example, result in the formation or depletion of gases at rates and magnitudes not observed in other environments^26–28^. These findings have drawn a generation of researchers to study this unique air-sea-ice environment and prompted us to expand the array of atmospheric trace gases monitored during the expedition.

Here, we present the comprehensive suite of *in-situ* surface trace gas measurements during the MOSAiC expedition, including CO_2_, CH_4_, N_2_O, O_3_, carbon monoxide (CO), dimethylsulfide (DMS), SO_2_, elemental mercury (Hg(0)), and selected volatile organic compounds (VOCs). Redundancy in certain measurements improved continuity and permitted cross-evaluation and validation of the measurements. We present the results of this intercomparison in an effort to demonstrate the quality of these individual datasets and of the overall trace gas monitoring activities during the expedition. In addition, we provide merged datasets (which combine redundant individual datasets and limit gaps in time series) for further use by the community.

## Methods

Anchored to an ice floe, the research icebreaker *Polarstern* drifted for an entire year over the central Arctic Ocean. The vessel departed from Tromsø, Norway on September 20, 2019. A suitable floe was found on October 4, 2019, at 85°N, 134°E, where the drift began. Due to logistical constraints related to the COVID-19 pandemic, *Polarstern* left the MOSAiC floe from mid-May to mid-June, 2020. Most measurements continued as the ship transited to Svalbard and back. From mid-June to the end of July, *Polarstern* was again attached to the MOSAiC floe. After the disintegration by melting and breakup of the original floe, the vessel transited to a new location close to the North Pole and drifted again from late August to late September, 2020. Most measurements continued as the ship transited back to Svalbard at the end of the expedition.

**Fig. 1:**
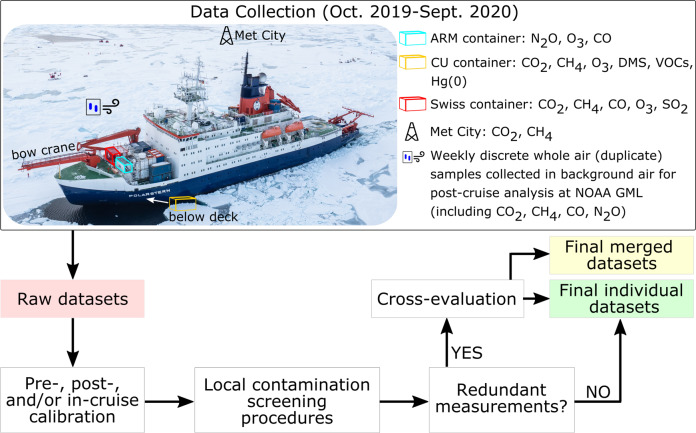
Experimental workflow. Trace gas ambient air measurements discussed in this paper were performed on sea ice, from a 10 m tower at Met City, and on-board *Polarstern* in three different sea-container laboratories, referred to as the Atmospheric Radiation Measurement (ARM; in blue), the University of Colorado (CU; in yellow), and Swiss (in red) containers. Note that instruments located in the CU container were connected to sampling inlets on the bow crane. Measurements included nitrous oxide (N_2_O), ozone (O_3_), carbon monoxide (CO), carbon dioxide (CO_2_), methane (CH_4_), dimethylsulfide (DMS), selected volatile organic compounds (VOCs), gaseous elemental mercury (Hg(0)), and sulfur dioxide (SO_2_). The post-cruise analysis of discrete whole air samples collected in background air (upwind from research activities) was performed at the National Oceanic and Atmospheric Administration (NOAA) Global Monitoring Laboratory (GML). Note that in addition to continuous DMS measurements, discrete samples were also occasionally collected for independent DMS analysis in the CU container. In case of redundant measurements (e.g., CO_2_), the cross-evaluated individual datasets were used to generate a merged dataset in order to limit gaps in time series and facilitate further use by the community. Photo credit: Jan Rohde.

**Fig. 2:**
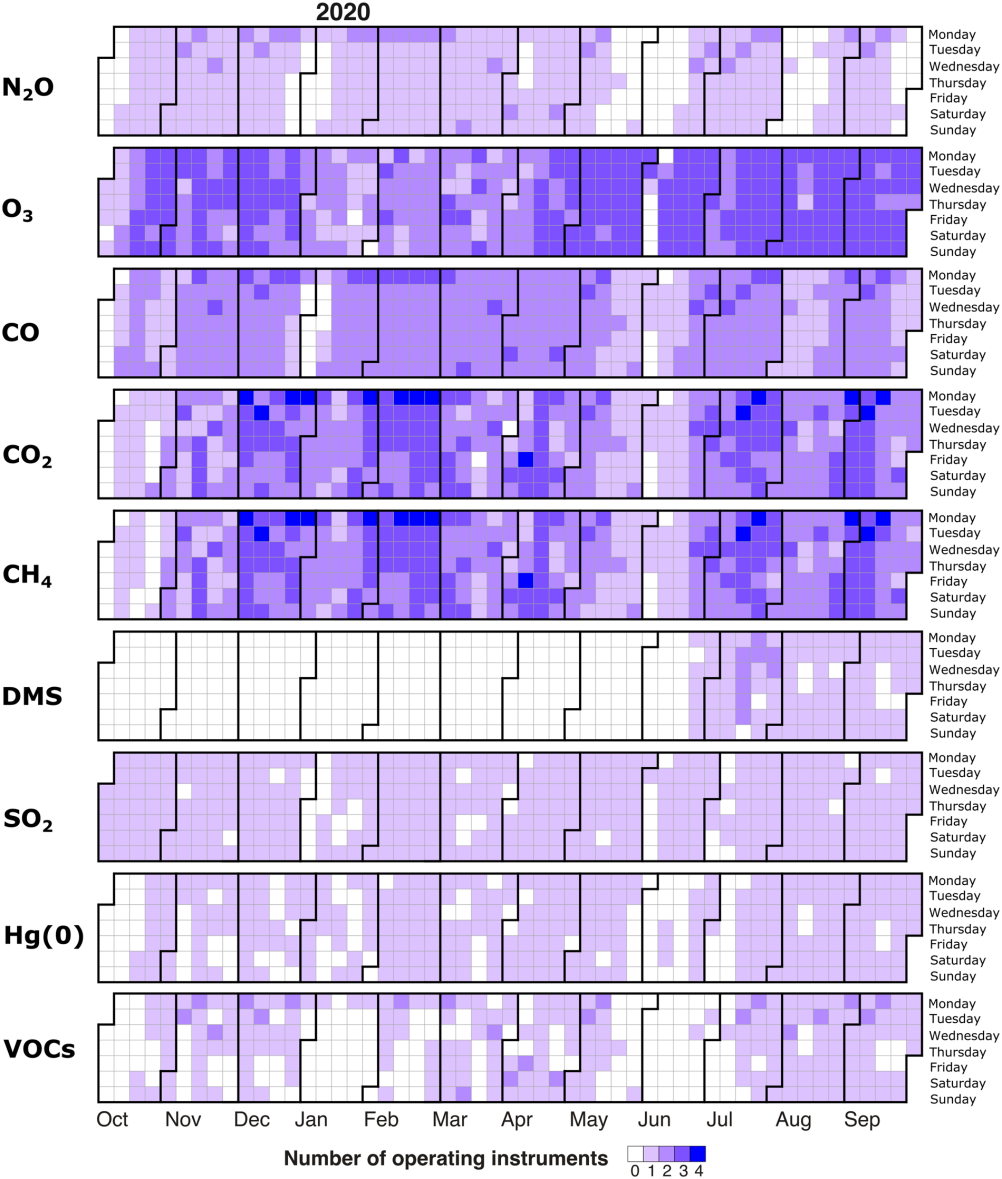
Number of operating instruments per day during the expedition. 0 (in white) indicates no measurements, either due to wind outside the clean air sector, ongoing maintenance operations, instrument failure, or when *Polarstern* was within Svalbard’s 12 nautical miles zone. Note that the archived individual datasets also include data collected when the wind was outside the clean air sector (in a separate column; see “Data Records” section).

As summarized in Fig. 1, trace gas measurements described hereafter were performed on-board *Polarstern* in three distinct sea-container laboratories, and on the sea ice itself from a 10-meter flux tower at Met City (meteorological station housing numerous atmospheric measurements located 300–600 m away from *Polarstern*^17^). While the Atmospheric Radiation Measurement (ARM) Program and Swiss containers were located on the foredeck (D-deck) with sampling inlets pointing upwards (inlet height of approximately 18 and 15 m above sea level (asl) in the ARM and Swiss containers, respectively), the University of Colorado (CU) container was installed below deck in the forward cargo hold. Sampling lines (roughly 50 m long) were deployed from the CU container to the bow crane to allow measurements forward of the vessel (Teflon lines for all instruments except a stainless-steel line for VOCs). The inlet height on the bow crane for slow trace gas measurements (Hg(0), O_3_, VOCs) was 15 m asl while inlets for the fast flux measurements (DMS, CO_2_, CH_4_) were at 20 m asl from October 2019 to May 2020, and 18 m asl for the rest of the expedition. Losses along the sampling lines are expected to be minimal for CO_2_, CH_4_, O_3_, and Hg(0). DMS losses are usually negligible but were accounted for by injecting internal standards at the inlet tip (see below). The temperature inside the different containers was kept constant at approximately 20 °C. Low ambient dew point temperatures (−20 to 0 °C) combined with the use of Nafion dryers limited the effect of water vapor on the measurements. The ARM container was operated as part of the United States Department of Energy (US DOE) Aerosol Observing System (AOS). As described by Uin *et al*.^29^, AOSs are designed as standardized platforms for atmospheric aerosol and trace gas measurements. The here reported trace gas measurements performed in the Swiss container were considered ancillary as the main objective was to monitor characteristics of aerosols and their precursors (see Fig. A3 in Beck *et al*.^30^ for a description of the full setup in this container). The comprehensive suite of *in-situ* trace gas measurements performed in the various containers during the expedition is summarized in Fig. 1 and Table 1. Figure 2 gives the number of operating instruments per day during the expedition.

**Table 1:** List of trace gas measurements discussed in this paper and associated instruments. Unless mentioned otherwise, measurements were continuous from October 2019 to September 2020.

Trace gas	Frequency	Time resolution	Sampling location	Instruments
CO_2_	Continuous	0.1 s	Met City	Cavity ring-down spectrometer (Picarro model G2311-f)
	Continuous	0.1 s	CU container	Cavity ring-down spectrometer (Picarro model G2311-f)
	Continuous	1 s	Swiss container	Cavity ring-down spectrometer (Picarro model G2401)
	Discrete	~weekly	Clean air sector	Cavity ring-down spectrometer
CH_4_	Continuous	0.1 s	Met City	Cavity ring-down spectrometer (Picarro model G2311-f)
	Continuous	0.1 s	CU container	Cavity ring-down spectrometer (Picarro model G2311-f)
	Continuous	1 s	Swiss container	Cavity ring-down spectrometer (Picarro model G2401)
	Discrete	~weekly	Clean air sector	Cavity ring-down spectrometer
CO	Continuous	1 s	ARM container	Off-axis integrated cavity output spectroscope (Los Gatos Research model 098-0014)
	Continuous	1 s	Swiss container	Cavity ring-down spectrometer (Picarro model G2401)
	Discrete	~weekly	Clean air sector	Tunable Infrared Laser Direct Absorption Spectroscopy (Aerodyne model CS-108)
N_2_O	Continuous	1 s	ARM container	Off-axis integrated cavity output spectroscope (Los Gatos Research model 098-0014)
	Discrete	~weekly	Clean air sector	Tunable Infrared Laser Direct Absorption Spectroscopy (Aerodyne model CS-108)
O_3_	Continuous	1 s	ARM container	Ultraviolet absorption spectroscopy (Thermo Fisher Scientific model 49i)
	Continuous	1 min	CU container	Ultraviolet absorption spectroscopy (Thermo Environmental Instruments model 49c)
	Continuous	10 s	Swiss container	Ultraviolet absorption spectroscopy (2B Technologies model 205)
DMS (June-Sept. 2020)	Continuous	0.1 s	CU container	Atmospheric Pressure Ionization Mass Spectrometer (custom fabrication)
	Discrete	3 hours	CU container	Gas chromatography with flame photometric detection (Shimadzu GC8/FPD)
SO_2_	Continuous	1 min	Swiss container	Pulsed fluorescence (Thermo Fisher Scientific model 43i)
Hg(0)	Continuous	15 min	CU container	Cold vapor atomic fluorescence spectrometer (Tekran model 2537B)
VOCs	Continuous	3 hours	CU container	*In-situ* air sampling with custom-build inlet system and gas chromatograph/mass spectrometry analysis
	Discrete	~weekly	Clean air sector	Mass spectrometry

See Fig. 1 for sampling locations. Note that instruments located in the CU container and at Met City were connected to sampling inlets on the 20-meter bow crane and 10-meter Met City tower, respectively.

### Continuous monitoring

#### Carbon dioxide, methane, carbon monoxide, and nitrous oxide

Atmospheric abundances, reported in dry air mole fractions, were monitored by cavity ring-down spectroscopy (CRDS) at Met City and in the CU and Swiss containers using commercial Picarro instruments (model G2311-f at Met City and in the CU container, model G2401 in the Swiss container; see Table 1). The Picarro instruments allow for simultaneous and continuous measurements of atmospheric trace gases along with water vapor. Dry air mole fractions were automatically obtained by applying water vapor correction factors^31^. The two G2311-f instruments were operated in 10 Hz flux mode during the expedition, with a manufacturer-specified precision <200 nmol/mol (parts per billion; ppb) for CO_2_ and <3 ppb for CH_4_. The G2401 instrument provided simultaneous measurements of CO_2_, CH_4_, and CO ambient air mole fractions, with a manufacturer-specified precision at 5 sec and 5 min of <50 ppb and 20 ppb for CO_2_, <1 ppb and 0.5 ppb for CH_4_, and <15 ppb and 1.5 ppb for CO. Simultaneous measurements of N_2_O, CO, and water vapor ambient air mole fractions were performed in the ARM container with an off-axis integrated cavity output spectroscopy instrument (OA-ICOS; Los Gatos Research model 098-0014) with a precision of 0.1 ppb for CO and 0.2 ppb for N_2_O^32^. Similarly to the Picarro instruments, the OA-ICOS instrument automatically corrects the measurements to dry conditions. Regular in-cruise calibrations were carried out for CO_2_, CH_4_, and CO to ensure the stability and accuracy of the response of the various instruments. The Picarro instrument in the Swiss container was calibrated using working standards that were characterized at the Swiss Federal Laboratories for Materials Science and Technology (EMPA) before the expedition. These working standards were directly calibrated against three standards traceable to the following calibration scales: WMO-X2007 for CO_2_^33^, WMO-X2004A for CH_4_^34^, and WMO-X2014A for CO^35^. The standards used at Met City and in the ARM and CU containers were working standards obtained from the Lawrence Berkley National Laboratory (ARM) and Airgas (Met City/CU). Due to logistical constraints before and after the expedition, these standards were not independently calibrated and are thus not traceable to the WMO calibration scales. Note that the OA-ICOS instrument was not calibrated for N_2_O as these measurements are considered ancillary by the US DOE AOS. Here, we report and compare minute-averaged ambient air mole fractions for all instruments.

#### Ozone

O_3_ ambient air mole fractions were monitored in the three afore-mentioned sea-container laboratories using commercial instruments (Thermo Fisher Scientific model 49i in the ARM container, Thermo Environmental Instruments model 49c in the CU container, and 2B Technologies model 205 in the Swiss container; see Table 1). These instruments have manufacturer-specified precisions of 1.0 ppb for 20-s averages. As described in detail in the instrument handbook^36^, the ARM instrument was checked twice a day for zero and span checks. Measurements during the zero measurement periods were used to calculate the instrument baseline with a 3–6 week moving average. This instrument baseline was then subtracted from the ambient air measurements. Measurements during zero and span checks were assessed for possible drifts. Note that measurements in the first 105 seconds after a zero and in the first 30 seconds after each span check were discarded. In addition, a linear calibration coefficient (determined from a five-point span check at the New York State Department of Environmental Conservation standards laboratory) was applied to O_3_ values. This final, quality checked, minute-averaged O_3_ dataset was used as reference to adjust O_3_ mole fractions from the Swiss and CU instruments. This is further discussed below in the sub-section “cross-evaluation of redundant measurements and merged datasets”. Note that zero and flow rate checks were performed every 2 weeks in the Swiss container.

#### Dimethylsulfide

Continuous DMS measurements were performed using an Atmospheric Pressure Ionization Mass Spectrometer with an Isotopically Labeled Standard (APIMS-ILS). The description of this custom-built instrument can be found in Appendix A of Blomquist *et al*.^37^. Briefly, the APIMS-ILS monitors the DMS mole fraction of a dried sample air stream at 10 Hz for analysis of the eddy correlation turbulent DMS flux. The air sample was drawn from an inlet at the top of the *Polarstern* bow sampling tower (Fig. 1), adjacent to a sonic anemometer. A known concentration of isotopically labeled DMS (d3-DMS, mass 65) was continuously injected at the inlet tip. The DMS mole fraction was computed from the signal intensity ratio of the protonated ambient and standard isotopomers (masses 63 and 66) and the gas flow rates. Note that the use of a continuous internal standard compensates for calibration drift and variable sensitivity. Averaged to 10 seconds, the APIMS-ILS detection limit is typically < 5 ppt. The d3-DMS compressed gas standard was calibrated with respect to a permeation tube device as the primary standard.

#### Sulfur dioxide

A commercial pulsed fluorescence instrument (Thermo Fisher Scientific model 43i) was used during the expedition with a flow rate of 0.5 L/min. Biweekly zero measurements were performed with a scrubber and we used an external permeation source to periodically check the calibration of the instrument during the expedition. This permeation source was characterized using a certified SO_2_ standard at the end of the expedition at EMPA. The instrument has a manufacturer-specified lower detectable limit of 1 ppb for a 1-minute averaging time.

#### Elemental mercury measurements

A Tekran 2537B mercury analyzer, commonly used at monitoring sites around the world^38–40^, was deployed in the CU container to monitor ambient air concentrations of Hg(0) during the expedition (see Fig. 1). To avoid potential bias in the default integration of the signal^41–43^, integrated samples were analyzed every 15 minutes. Millipore 0.45 µm polyether sulfone cation-exchange membranes were used to remove potential divalent Hg species, and thus, only Hg(0) was collected and analyzed here^44–46^. The instrument was automatically calibrated every 25 hours using an internal Hg permeation source. The accuracy of the permeation source was checked before the beginning of the expedition against manual injections of saturated Hg vapor using a Tekran 2505 Hg vapor calibration unit^47^. Screening criteria for data validation/invalidation were inspired by standard operative protocols used by the Canadian Atmospheric Mercury Measurement Network (CAMNet), the US Atmospheric Mercury Network (AMNet), and the Global Mercury Observation System (GMOS) network^48,49^. The average systematic uncertainty for Hg(0) measurements is of approximately 10% based on experimental evidence^50^.

#### Volatile organic compounds

An automated gas chromatography and mass spectrometry with flame ionization detector (GC-MS/FID) system was used for continuous measurements of selected VOCs at a 3-hr time resolution. Ambient air, pulled from the inlet on the bow crane, passed through a u-shaped Silcosteel^TM^ (stainless steel treated) moisture trap cooled with thermoelectric coolers to dry the air to a dew point of −45 °C, and through a sodium thiosulfate-coated O_3_ scrubber to prevent sampling losses and artifacts^51^. Analytes were concentrated on a Peltier-cooled (−40 °C) multistage micro-adsorbent trap (Carboxen 569 and Carboxen 1000). Analysis was performed by thermal desorption and injection for cryogen-free GC using a Porabond-Q column (50 m × 320 µm × 5 µm) and helium as a carrier gas. Blanks and calibration standards were regularly injected from a manifold. In order to monitor and correct for trends in the detection system (i.e., detector drift, decreasing performance of the adsorbent trap), we used peak areas of long-lived chlorofluorocarbons (CFCs) that were monitored in the air samples together with VOCs as an internal reference standard^52,53^. Table 2 gives the full list of compounds included in the selected ion-monitoring (SIM) mode target list. We only report here mole fractions for a subset of compounds (propane, isobutane, n-butane, and isoprene); We welcome enquiries regarding the quantification of other compounds listed in Table 2 or the identification of compounds not listed here (SCAN mode chromatograms). Please note that the raw chromatograms are also publicly available on the Arctic Data Center repository (see Data Records section). Propane, isobutane, n-butane, and isoprene were identified and quantified using the MS in SIM mode and a UK-National Physical Laboratory (NPL) calibration standard. The repeatability of these measurements was estimated to 5–6% based on the repeated analysis (n = 54) of the NPL standard throughout the expedition. Chromatograms were analyzed using the TERN (Thermal desorption aerosol gas chromatography ExploreR and iNtegration package) peak fitting tool^54^.

**Table 2:** List of compounds included in the GC-MS/FID Selected Ion-Monitoring (SIM) target list.

Target compounds	Quantifying ion
1-Chloro-1,1-difluoroethane (HCFC-142b)	65
1-Chloro-1,2,2,2-tetrafluoroethane (HCFC-124)	67
1,1-Dichloro-1-fluoroethane (HCFC-141b)	81
1,1-Difluoroethane (HFC-152a)	65
1,1,1,2-Tetrafluoroethane (HFC-134a)	83
1,1,2-Trichloro-1,2,2-trifluoroethane (CFC-113)	151
1,2-Dichlorotetrafluoroethane (CFC-114)	135
Acetaldehyde (C_2_H_4_O)	43
Acetone (C_3_H_6_O)	58
Benzene (C_6_H_6_)	78
Bromochlorodifluoromethane (H-1211)	85
Bromochloromethane (CH_2_BrCl)	128
Bromodichloromethane (CHBrCl_2_)	83
Bromoform (CHBr_3_)	173
Bromomethane (CH_3_Br)	94
Butane (C_4_H_10_)	43
Carbon disulfide (CS_2_)	76
Carbon tetrachloride (CCl_4_)	121
Carbonyl sulfide (OCS)	60
Chlorodifluoromethane (HCFC-22)	67
Chloroform (CHCl_3_)	83
Chloromethane (CH_3_Cl)	50
Dibromomethane (CH_2_Br_2_)	174
Dichlorodifluoromethane (CFC-12)	85
Dichloromethane (CH_2_Cl_2_)	84
Ethane (C_2_H_6_)	27
Iodomethane (CH_3_I)	142
Isobutane (C_4_H_10_)	43
Isopentane (C_5_H_12_)	43
Isoprene (C_5_H_8_)	68
Pentafluoroethane (HFC-125)	101
Pentane (C_5_H_12_)	43
Perchloroethylene (C_2_Cl_4_)	166
Propane (C_3_H_8_)	43
Propyne (C_3_H_4_)	40
Sulfur hexafluoride (SF_6_)	89
Toluene (C_7_H_8_)	92
Trichlorofluoromethane (CFC-11)	101

### Discrete monitoring

#### Global Greenhouse Gas Reference Network

Whole air samples were collected ~weekly following established protocols of the National Oceanic and Atmospheric Administration (NOAA) Global Monitoring Laboratory (GML) Carbon Cycle Cooperative Global Air Sampling Network (https://gml.noaa.gov/ccgg/). Samples were collected in pairs, in background air, upwind from local emissions, in 2.5 L borosilicate flasks with two glass-piston stopcocks sealed with Teflon O-rings. Flasks were flushed in series for 5 minutes then pressurized to ~1.2 atm with a portable sampling system^55,56^. Samples were transported back to the NOAA GML facility in Colorado, United States and analyzed post-cruise following well-established protocols. CO_2_ and CH_4_ were analyzed by cavity ring-down spectroscopy while N_2_O and CO were analyzed using a Tunable Infrared Laser Direct Absorption Spectroscopy (TILDAS) method. The analyzers are routinely calibrated off-line once a month with a suite of standards. The repeatability of the measurements is estimated to be 0.02 *μm*ol/mol (parts per million; ppm) for CO_2_, 0.2 ppb for CH_4_, 0.02 ppb for N_2_O, and 0.1 ppb for CO based on the repeated analysis of air from a high-pressure cylinder. All measurements were referenced to the corresponding NOAA calibration scales (https://gml.noaa.gov/ccl/scales.html), i.e., the World Meteorological Organisation (WMO) X2019 CO_2_ scale^57^, the WMO-X2004A CH_4_ standard scale^34^, the NOAA-2006A N_2_O standard scale^58^, and the WMO-X2014A CO scale^35^. In addition to CO_2_, CH_4_, CO, and N_2_O, samples were also analyzed for other hydrocarbons under the umbrella of the NOAA GML Halocarbons and other Atmospheric Trace Species (HATS) network (https://gml.noaa.gov/hats/) and for stable isotopes of CO_2_ and CH_4_ at the University of Colorado Institute for Arctic and Alpine Research^59,60^. Table 3 gives the full list of measurements performed on the discrete whole air samples collected during MOSAiC.

**Table 3:** List of post-cruise measurements performed on the weekly whole air discrete samples.

Trace gas
Methane (CH_4_)
Stable isotopes of methane (δ^13^C)
Carbon monoxide (CO)
Carbon dioxide (CO_2_)
Stable isotopes of carbon dioxide (δ^13^C, δ^18^O)
Molecular hydrogen (H_2_)
Nitrous oxide (N_2_O)
Sulfur hexafluoride (SF_6_)
Acetylene (C_2_H_2_)
Ethane (C_2_H_6_)
Propane (C_3_H_8_)
Isobutane (C_4_H_10_)
Isopentane (C_5_H_12_)
n-Butane (C_4_H_10_)
n-Pentane (C_5_H_12_)
n-Hexane (C_6_H_14_)
Benzene (C_6_H_6_)

#### Dimethylsulfide

To complement the APIMS-ILS measurements, ambient DMS mole fractions were intermittently measured using an automated gas chromatography and flame photometric detector (GC/FPD) system. DMS in air pulled from the inlet on the bow crane was automatically sampled over a period of 45 minutes at a flow rate of 0.200 L/min, and concentrated on adsorbent tubes containing a mixture of Carboxen 1016 and Carboxen 1000, held at 30 °C. An automatic thermal desorption system (PerkinElmer ATD 400) was used to transfer samples to a GC (Shimadzu GC8/FPD) with a Chromosil 330 packed column (4 m × 2.1 mm), using helium as the carrier gas. Calibration made use of the same DMS standard (d3-DMS, 576 ppb) as used as the internal standard in the APIMS-ILS system.

### Local pollution screening procedures

While frozen into the pack ice, it was not possible to maneuver the ship’s bow into the prevailing wind for clean air sampling, so all measurements were episodically influenced by local anthropogenic pollution sources (e.g., exhaust by the vessel’s engine and vents, skidoos, helicopters, on-ice diesel generators). Different screening strategies were employed in the three sea-container laboratories to identify and/or mitigate these influences. Sampling of polluted air was prevented in the CU container by automatically backflushing the inlet stack with zero-air during unfavorable wind conditions, empirically determined to be a relative wind direction more than ± 130° from the bow. Similarly, for the Met City measurements, true wind direction within ± 10° of the compass bearing from the tower to the ship was excluded. The ARM container was equipped with a purge blower set up to trigger based on ambient CO mole fractions. As CO turned out not to be an ideal tracer for local pollution from the ship stack of *Polarstern* during MOSAiC^30^, the purge blower was turned on manually when the container was exposed to local pollution for extended periods, as identified by local operators. The purge blower only affected the O_3_ data, as the CO analyzer was sampling from a separate inlet line collocated with the main AOS inlet. Lastly, sampling was uninterrupted in the Swiss container. As a result, all datasets were carefully screened for local pollution during post processing as described hereafter.

As described in Beck *et al*.^30^, local pollution typically leads to rapid fluctuations in measurements of many parameters. Local pollution in such a remote environment can often be detected based on the time derivative of the ambient air mole fraction. For each data point, the time derivative was calculated. Data points corresponding to an abnormally high derivative (>1.5 times the interquartile range) and neighboring points were discarded. The function “despike” from R package oce^61^ (version 1.3-0) was then applied to the time-series to remove any remaining local pollution spikes. Briefly, this function first linearly interpolates across any gaps (missing values). Then, it calculates a running median spanning k elements. The result of these two steps is the “reference” time-series. The standard deviation of the difference between values and the reference is then calculated. Values that differ from the reference by more than n times this standard deviation are considered to be spikes and eliminated. The function was applied once with n = 1 (n = 3 for CO^62^) and k = 61 (~1 hour). This procedure was applied to all datasets, unless mentioned otherwise below.

As the time derivative method is better suited for primary pollutants^30^, O_3_ time-series were cleaned from local pollution influence using the above-mentioned “despike” function only. The function was applied twice using different k values (k = 1439 (~1 day) and k = 61 (~1 hour)) and n = 3, which satisfactorily eliminated negative O_3_ spikes due to local nitric oxide emissions.

In parallel, CO_2_ and CH_4_ time-series collected in the CU container and at Met City exclude all measurements that were not in the clean wind sector (see above), eliminating the majority of local pollution events. Selected instances of emission spikes from equipment operations on-ice during working hours (e.g., skidoos) were identified and removed manually.

### Cross-evaluation of redundant measurements and creation of merged datasets

Redundant measurements were cross-evaluated using i) weekly discrete samples collected for post-cruise analysis at NOAA GML as calibration reference for CO_2_, CH_4_, and CO, and ii) ARM measurements as calibration reference for O_3_. Ambient air mole fractions were adjusted with respect to reference measurements using the slope and intercept of the correlation with initial (non-adjusted) values (Eq. 1).

1 $$adjusted\;mole\;fraction=\frac{initial\;mole\;fraction-intercept}{slope}$$

**Fig. 3:**
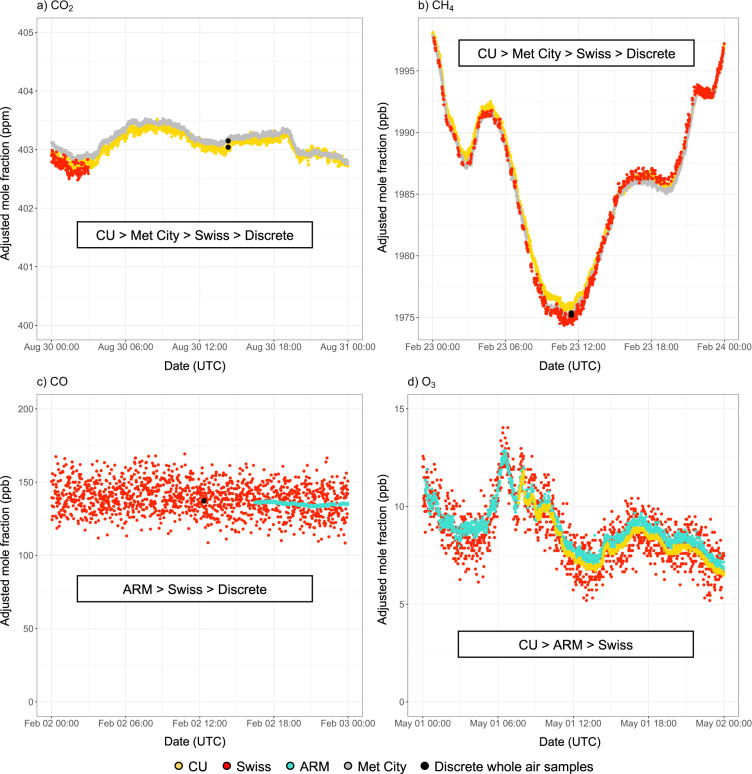
Creation of merged datasets. Order of priority, based on the precision and frequency of the measurements, given to the different cross-evaluated individual datasets for the creation of the merged datasets. This Figure shows minute-averaged adjusted (after cross-evaluation) time-series collected in the three containers (CU, Swiss, and ARM) and at Met City, along with discrete whole air samples collected for post-cruise analysis.

Time-dependent correction factors (slope and intercept) were used to account for drifts in differences between instruments. Correction factors can be found in Tables 4 and 5. The cross-evaluated adjusted mole fractions were then used to generate hourly-averaged merged datasets in order to limit gaps in the time series and facilitate use by the scientific community. Figure 3 shows the order of priority given to the different cross-evaluated individual datasets for the creation of the hourly-averaged merged datasets. Priority was given to continuous measurements over discrete samples, and to instruments with the highest precision. As summarized in Table 1, the cavity ring-down instruments used in the CU container and at Met City are designed for flux measurements and were operated in 10 Hz mode. This resulted in higher precision minute-averaged measurements of CO_2_ and CH_4_ as compared to measurements in the Swiss container. Priority was thus given to these two individual datasets for the creation of the merged datasets. Following a similar approach, priority was given to CO measurements performed in the ARM container (manufacturer-specified precision at 1 sec of 0.1 ppb for the OA-ICOS instrument) over measurements performed in the Swiss container (manufacturer-specified precision at 5 sec < 15 ppb for the G2401 instrument). Finally, priority was given to the O_3_ measurements performed in the CU container over measurements performed in the ARM and Swiss containers (Fig. 3d). Figure 3 also highlights the very good agreement between the different adjusted time-series, reflecting the high quality of the monitoring activities during the expedition. This is further discussed in the “Technical Validation” section.

**Table 4:** Correction factors.

Trace gas	Sampling location	October to February		March to June		July to September	
		slope	intercept	slope	intercept	slope	intercept
CH_4_	Swiss container	0.9767	45.2709	1.0062	−12.6131	1.0240	−47.2318
	CU container	1.0095	−10.7082	1.0712	−132.0740	0.9407	122.5348
	Met City	0.9856	31.3075	0.9856	31.3075	0.9856	31.3075
CO_2_	Swiss container	1.0333	−13.4394	0.9753	10.1641	0.9364	25.2725
	CU container	0.9957	−0.0138	1.0993	−43.8172	0.9696	9.8459
	Met City	0.9943	0.07443	0.9943	0.07443	0.9943	0.07443
O_3_	Swiss container	0.6343	7.6929	0.7455	1.2322	0.6581	4.2044
	CU container	0.8217	4.1540	0.9185	0.6376	0.7865	4.8725

Correction factors (slope and intercept) applied to CO_2_, CH_4_, and O_3_ measurements in the CU and Swiss containers and at Met City. Weekly discrete samples collected for post-cruise analysis at NOAA GML are used as calibration reference for CO_2_, and CH_4_ measurements while ARM measurements are used as calibration reference for O_3_ measurements. The intercept is given in ppb for CH_4_ and O_3_, and in ppm for CO_2_.

**Table 5:** Correction factors.

October to December		January to March		April to June		July to September	
slope	intercept	slope	intercept	slope	intercept	slope	intercept
1.0384	−18.8750	0.3607	69.5694	0.9544	−19.3850	0.5107	21.8390

Correction factors (slope and intercept (in ppb)) applied to CO measurements in the Swiss container. Weekly discrete samples collected for post-cruise analysis at NOAA GML are used as calibration reference.

## Data Records

Table 6 summarizes data records associated with this work, including the repositories where data are stored. Tables 7 and 8 summarize the list of attributes for datasets collected in the Swiss container and for merged datasets, respectively, both archived on PANGAEA (https://www.pangaea.de/). Datasets collected in the CU container and at Met City are archived on the Arctic Data Center (https://arcticdata.io/); the list of attributes can be found in Table 9. Datasets collected in the ARM container are archived on the ARM Data Archive (https://www.arm.gov/data/); the list of attributes can be found in Tables 10 and 11. Finally, data inferred from discrete whole air sampling and post-cruise analysis at NOAA GML are available on a dedicated webpage (https://gml.noaa.gov/ccgg/arc/?id=157); the list of attributes can be found in Table 12. Please note that one needs to register to access datasets archived on the ARM Data Archive and at NOAA GML and that contact information will be sent to contributing data providers.

**Table 6:** Data records.

Trace gas	Type	Time resolution	Sampling location	Calibration scale	Data repository
CO_2_	Individual	1 min	Met City	WMO-X2019	Blomquist *et al*.^66^
	Individual	1 min	CU container	WMO-X2019	Blomquist *et al*.^67^
	Individual	1 min	Swiss container	WMO-X2019	Angot *et al*.^68^
	Individual	~weekly	Clean air sector	WMO-X2019	Dlugokencky *et al*.^69^
	Merged	1 hour	Multiple	WMO-X2019	Angot *et al*.^70^
CH_4_	Individual	1 min	Met City	WMO-X2004A	Blomquist *et al*.^71^
	Individual	1 min	CU container	WMO-X2004A	Blomquist *et al*.^72^
	Individual	1 min	Swiss container	WMO-X2004A	Angot *et al*.^73^
	Individual	~weekly	Clean air sector	WMO-X2004A	Dlugokencky *et al*.^69^
	Merged	1 hour	Multiple	WMO-X2004A	Angot *et al*.^74^
CO	Individual	1 s	ARM container		Trojanowski and Springston^75^
	Individual	1 min	Swiss container	WMO-X2014A	Angot *et al*.^76^
	Individual	~weekly	Clean air sector	WMO-X2014A	Dlugokencky *et al*.^69^
	Merged	1 hour	Multiple	WMO-X2014A	Angot *et al*.^77^
N_2_O	Individual	1 s	ARM container		Trojanowski and Springston^75^
	Individual	~weekly	Clean air sector	NOAA-2006A	Dlugokencky *et al*.^69^
O_3_	Individual	1 s	ARM container		Springston and Koontz^78^
	Individual	1 min	CU container		Angot *et al*.^79^
	Individual	1 min	Swiss container		Angot *et al*.^80^
	Merged	1 hour	Multiple		Angot *et al*.^81^
DMS	Individual	1 min	CU container		Blomquist *et al*.^82^
SO_2_	Individual	1 min	Swiss container		Angot *et al*.^83^
Hg(0)	Individual	30 min	CU container		Angot *et al*.^84^
VOCs	Individual	3 hours	CU container		Angot *et al*.^85^
					Raw chromatograms: Angot *et al*.^86^
	Discrete	~weekly	Clean air sector		Dlugokencky *et al*.^69^

See Fig. 1 for sampling locations. Merged datasets combine cross-evaluated individual datasets collected onboard *Polarstern* for further use by the community. Cautionary note: ARM datasets available online are not screened for local pollution. They were, however, screened for local pollution in this analysis before use in merged datasets. In addition, please note that the ARM N_2_O measurements were not calibrated; we recommend the use of the NOAA discrete N_2_O dataset instead. Final cross-evaluated redundant measurements of CO_2_, CH_4_, and CO are all traceable to the NOAA/WMO calibration scales.

**Table 7:** List of attributes in the files originating from the Swiss container and archived on PANGAEA.

Variable	Definition
Date_Time_UTC	Date and Time of measurement in Coordinated Universal Time and ISO-format (YYYY-MM-DDThh:mm:ss)
latitude	Latitude of Research Vessel Polarstern in degrees north
longitude	Longitude of Research Vessel Polarstern in degrees east
MOSAiC_event_label	Event list of MOSAiC campaign PS122
compound_unit *(CO_ppb, CO2_ppm, CH4_ppb, O3_ppb or SO2_ppb)*	Minute-averaged initial ambient air mole fraction
compound_unit_adjusted *(CO_ppb_adjusted, CO2_ppm_adjusted, CH4_ppb_adjusted or O3_ppb_adjusted)*	Minute-averaged adjusted ambient air mole fraction after cross-evaluation
pollution_flag	Pollution flag were “yes” means that local pollution was detected
detection_limit_flag	Detection limit flag where “yes” indicates that the mole fraction was below the lower detectable limit

The variable *detection_limit_flag* is only present for sulfur dioxide.

**Table 8:** List of attributes in the merged datasets archived on PANGAEA.

Variable	Definition
Date_Time_UTC	Date and Time of measurement in Coordinated Universal Time and ISO-format (YYYY-MM-DDThh:mm:ss)
latitude	Latitude of Research Vessel Polarstern in degrees north
longitude	Longitude of Research Vessel Polarstern in degrees east
MOSAiC_event_label	Event list of MOSAiC campaign PS122
merged_compound_unit *(merged_CO_ppb, merged_CO2_ppm, merged_CH4_ppb or merged_O3_ppb)*	Hourly-averaged adjusted ambient air mole fraction after cross-evaluation, exempt from local anthropogenic pollution
sampling_location	Identifies the location where the measurement was performed (e.g., Swiss container, CU container, ARM container, Met City, Discrete sampling).

**Table 9:** List of attributes in the files originating from the CU container or Met City, and archived on the Arctic Data Center.

Variable	Definition
Date_Time_UTC	Date and Time of measurement in Coordinated Universal Time
latitude	Latitude of Research Vessel Polarstern in degrees north
longitude	Longitude of Research Vessel Polarstern in degrees east
MOSAiC_event_label	Event list of MOSAiC campaign PS122
compound_unit *(CH4_ppb, CO2_ppm, O3_ppb, DMS_ppt, Hg0_ng_per_m3, propane_ppt, i_butane_ppt, n_butane_ppt, isoprene_ppt)*	Ambient air mole fraction or concentration
compound_unit_adjusted (*CH4_ppb_adjusted, CO2_ppm_adjusted, O3_ppb_adjusted*)	Adjusted ambient air mole fraction after cross-evaluation
pollution_flag	Pollution flag were “yes” means that local pollution was detected

The variable *compound_unit_adjusted* is present only in case of cross-evaluation. As measurements were only performed when the wind was from the clean air sector, the files do not include a pollution flag (data exempt from local anthropogenic pollution). The variable *pollution_flag* is only present for ozone for which a few data points had to be manually flagged.

**Table 10:** List of attributes in the files originating from the ozone instrument in the ARM container, and archived on the ARM Data Archive.

Variable	Definition
time	Date and Time of measurement in Coordinated Universal Time.
base_time	Base time (“2019-10-11 00:00:00” in our case).
time_offset	Time offset from base time.
o3	Ozone concentration at standard temperature and pressure in parts per billion. Missing values are denoted −9999.
qc_o3	Quality control flag. This field contains bit packed integer values, where each bit represents a QC test on the data. Non-zero bits indicate the QC condition given in the description for those bits (see file *mosaoso3M1.b1.20191011.000000.header.txt* for a full description); a value of 0 (no bits set) indicates the data has not failed any QC tests.
time_of_last_state_change	Time of last state change.
o3_pressure	Ozone pressure in kPa.
o3_bench_temperature	Bench temperature in degrees Celsius.
o3_lamp_temperature	Ozone lamp temperature in degrees Celsius.
flow_a	Flow in cell A in L/min.
flow_b	Flow in cell B in L/min.
noise_a	Electric noise in cell A in Hz.
noise_b	Electric noise in cell B in Hz.
averaging_time	Instrument averaging time in seconds.
intensity_a	Intensity in cell A in Hz.
intensity_b	Intensity in cell B in Hz.
lamp_temperature	Lamp temperature in degrees Celsius.
lamp_voltage_bench	Lamp bench voltage in volts.
lamp_voltage_ozonizer	Lamp voltage of ozonizer in volts.
lamp_level	Lamp level in %.
range	Instrument range setting in parts per billion.
o3_coefficient	Instrument ozone coefficient.
o3_background	Ozone background in parts per billion.
calibration_level_1 to _5	Calibration levels.
pressure_compensation_state	Pressure compensation state.
temperature_compensation_state	Temperature compensation state.
o3_lamp_state	Ozone lamp state.
gas_state	Gas state.
diagnostic_voltage_mb_24	Diagnostic + 24 volts at motherboard.
diagnostic_voltage_mb_15	Diagnostic + 15 volts at motherboard.
diagnostic_voltage_mb_5	Diagnostic + 5 volts at motherboard.
diagnostic_voltage_mb_3p3	Diagnostic + 3.3 volts at motherboard.
diagnostic_voltage_mb_minus_3p3	Diagnostic −3.3 volts at motherboard.
diagnostic_voltage_mib_24	Diagnostic + 24 volts at measurement interface board.
diagnostic_voltage_mib_15	Diagnostic + 15 volts at measurement interface board.
diagnostic_voltage_mib_minus_15	Diagnostic −15 volts at measurement interface board.
diagnostic_voltage_mib_5	Diagnostic + 5 volts at measurement interface board.
diagnostic_voltage_mib_3p3	Diagnostic + 3.3 volts at measurement interface board.
o3_flags	Ozone flag string.
o3_offset	Offset used in O_3_ correction in parts per billion.

**Table 11:** List of attributes in the files originating from the off-axis integrated cavity output spectroscopy instrument in the ARM container, and archived on the ARM Data Archive.

Variable	Definition
time	Date and Time of measurement in Coordinated Universal Time.
base_time	Base time (“2019-10-11 00:00:00” in our case).
time_offset	Time offset from base time.
seconds_after_calibration	Seconds after calibration.
co	Carbon monoxide mixing ratio calculated with nominal sensitivity correction in parts per million.
qc_co	Quality control flag. This field contains bit packed integer values, where each bit represents a QC test on the data. Non-zero bits indicate the QC condition given in the description for those bits (see file *mosaoscoM1.b1.20191011.004433.header.txt* for a full description); a value of 0 (no bits set) indicates the data has not failed any QC tests.
n2o	Nitrous oxide mixing ratio calculated with nominal sensitivity correction in parts per million.
qc_n2o	Quality control flag. This field contains bit packed integer values, where each bit represents a QC test on the data. Non-zero bits indicate the QC condition given in the description for those bits (see file *mosaoscoM1.b1.20191011.004433.header.txt* for a full description); a value of 0 (no bits set) indicates the data has not failed any QC tests.
h2o	Water vapor mixing ratio calculated with nominal sensitivity correction in parts per million.
qc_h2o	Quality control flag. This field contains bit packed integer values, where each bit represents a QC test on the data. Non-zero bits indicate the QC condition given in the description for those bits (see file *mosaoscoM1.b1.20191011.004433.header.txt* for a full description); a value of 0 (no bits set) indicates the data has not failed any QC tests.
co_dry	Carbon monoxide mixing ratio corrected for water vapor concentration and calculated with nominal sensitivity correction in parts per million.
qc_co_dry	Quality control flag. This field contains bit packed integer values, where each bit represents a QC test on the data. Non-zero bits indicate the QC condition given in the description for those bits (see file *mosaoscoM1.b1.20191011.004433.header.txt* for a full description); a value of 0 (no bits set) indicates the data has not failed any QC tests.
n2o_dry	Nitrous oxide mixing ratio corrected for water vapor concentration and calculated with nominal sensitivity correction in parts per million.
qc_n2o_dry	Quality control flag. This field contains bit packed integer values, where each bit represents a QC test on the data. Non-zero bits indicate the QC condition given in the description for those bits (see file *mosaoscoM1.b1.20191011.004433.header.txt* for a full description); a value of 0 (no bits set) indicates the data has not failed any QC tests.
gas_pressure	Cell sample pressure in torr.
gas_temperature	Cell sample temperature in degrees Celsius.
ambient_temperature	Ambient temperature of instrument in degrees Celsius.
set_point_for_MFC_1	Set point for mass flow controller 1 in cm^3^/min.
mass_flow_through_MFC_1	Actual mass flow through mass flow controller 1 in cm^3^/min.
valve_position_MFC_1	Valve position for mass flow controller 1 (0 for open, 1 for closed, −1 for error).
set_point_for_MFC_2	Set point for mass flow controller 2 in cm^3^/min.
mass_flow_through_MFC_2	Actual mass flow through mass flow controller 2 in cm^3^/min.
valve_position_MFC_2	Valve position for mass flow controller 2 (0 for open, 1 for closed, −1 for error).

**Table 12:** List of attributes in the files originating from discrete whole air samples, and archived on the NOAA GML webpage.

Variable	Definition
sample_site_code	Three-character sampling location code (“CRS” for “cruise” in our case).
sample_year	The sample collection date and time in Coordinated Universal Time.
sample_month	
sample_day	
sample_hour	
sample_minute	
sample_seconds	
sample_id	The sample container ID
sample_method	A single-character code that identifies the sample collected method (“P” on our case, meaning that the sample was collected using a portable, battery powered pumping unit – See methods section).
parameter_formula	Gas identifier (e.g., CO_2_, C_2_H_6_).
analysis_group_abbr	Identifies the group with NOAA and INSTAAR making the actual measurement (e.g., CCGG, HATS, SIL).
analysis_value	Dry air mole fraction or isotopic composition. Missing values are denoted by −999.99.
analysis_uncertainty	Estimated uncertainty of the reported measurement value. Missing values are denoted −999.99.
analysis_flag	A three-column quality control flag indicating the results of data rejection and selection process. Column 1: rejection flag. An alphanumeric other than a period (.) in the first column indicates a sample with obvious problems during collection or analysis. This measurement should not be interpreted. Column 2: selection flag. An alphanumeric other than a period (.) in the second column indicates a sample that is likely valid but does not meet selection criteria determined by the goals of a particular investigation. Column 3: information flag. An alphanumeric other than a period (.) in the third column provides additional information about the collection or analysis of the sample. Note that a “P” in the third column indicates the measurement result is preliminary.
analysis_instrument	A 2-character code that identifies the instrument used for the measurement.
analysis_year	The measurement date and time in local time.
analysis_month	
analysis_day	
analysis_hour	
analysis_minute	
analysis_seconds	
sample_latitude	The latitude where the sample was collected in degrees north.
sample_longitude	The longitude where the sample was collected in degrees east.
sample_altitude	The altitude of the sample inlet in meters above sea level. The reported altitude is the surface elevation plus sample intake height.
sample_elevation	Surface elevation in meters above sea level.
sample_intake_height	Air sample collection height above ground level.
event_number	A long integer that uniquely identifies the sampling event.

As measurements were only performed when the wind was from the clean air sector, the files do not include a pollution flag (data exempt from local anthropogenic pollution).

## Technical Validation

**Fig. 4:**
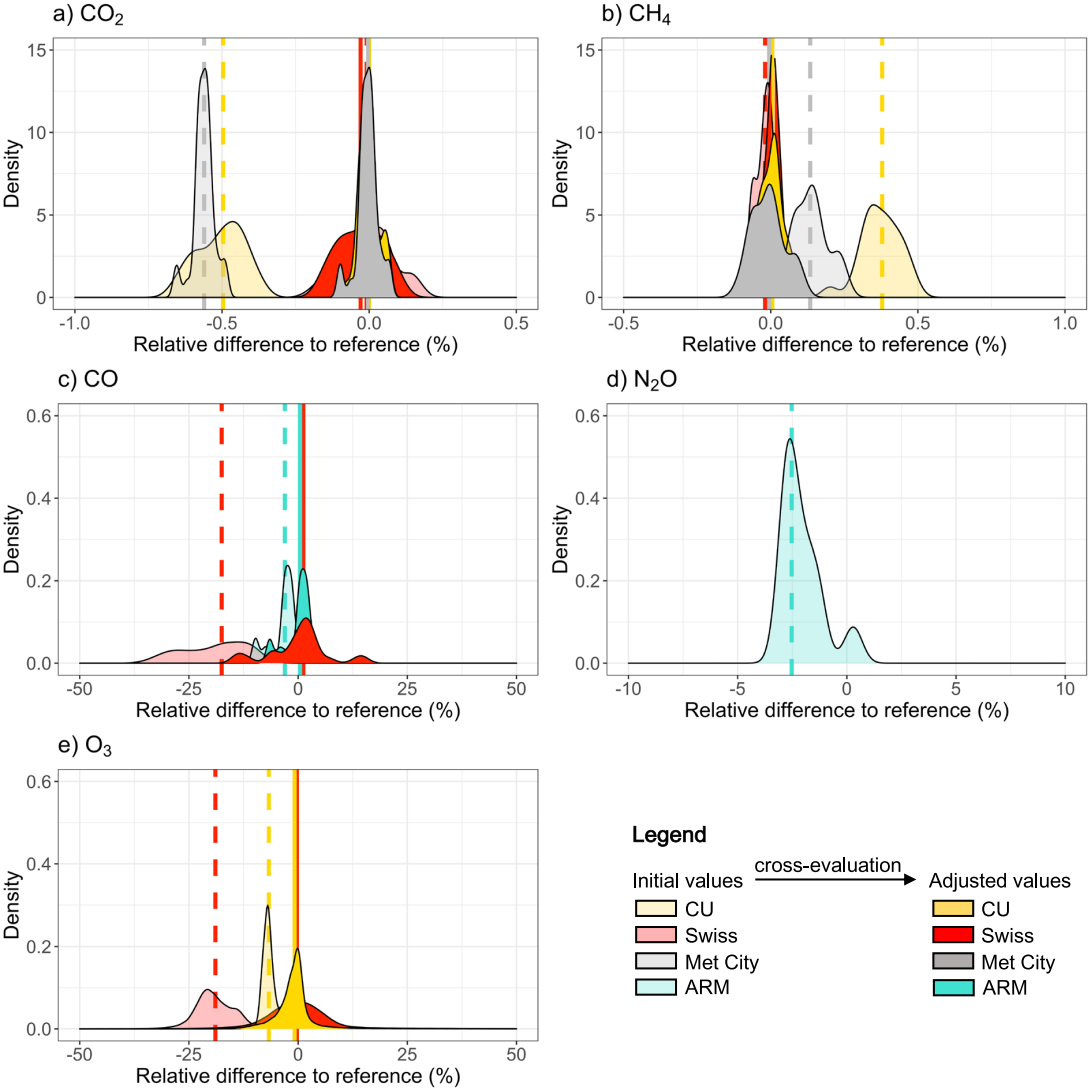
Cross-evaluation of redundant measurements. Comparison of minute-averaged (**a**) CO_2_, (**b**) CH_4_, (**c**) CO and (**d**) N_2_O mole fractions measured in the University of Colorado (CU), Swiss, and Atmosphere Radiation Measurement (ARM) Program containers against atmospheric abundances inferred from discrete whole air sampling for post-cruise analysis at NOAA Global Monitoring Laboratory (used as calibration reference). (**e**) Comparison of minute-averaged O_3_ mole fractions measured in the CU and Swiss containers against mole fractions measured in the ARM container (used as calibration reference). Shaded and solid contours show initial and adjusted (after cross-evaluation) values, respectively. The shape of the density distributions may change due to the use of time-dependent correction factors. Vertical dashed and solid lines show the median relative difference to the reference for initial and adjusted values, respectively. Note that the ARM N_2_O time series was not adjusted for calibration bias as we did not generate a merged N_2_O dataset. The kernel density estimates (smoothed version of a histogram) were computed using R package ggplot2 (version 3.3.3).

The comparison of redundant measurements before and after cross-evaluation are presented in Fig. 4. Redundant measurements were performed using completely independent setups (inlet, instrument, calibration standards) and biases are thus expected. The CU CO_2_ and CH_4_ measurements were for instance biased low (median relative difference to the NOAA GML reference of −0.50%) and high (+0.38%), respectively. The Swiss CO and O_3_ time series were biased low (−17.5% and −19.0%, respectively, relative to CO discrete samples and ARM O_3_ data) while the CU O_3_ time series was biased low by −6.7% relative to ARM O_3_ data. The median relative differences between redundant measurements of CO_2_, CH_4_, and CO were relatively large (larger than the WMO compatibility guidelines, see below). These differences can largely be explained by the use of different working standards that were not all traceable to the same calibration scale (see Methods section). The cross-evaluation step allows for correction of these calibration biases. In addition, the use of time-dependent correction factors (see above) removes biases associated with the potential drift of instruments. After the cross-evaluation step, redundant measurements are now fully consistent. For instance, on average over the full period, the difference between hourly-averaged continuous measurements is 0.08 ppm for CO_2_ and 0.06 ppb for CH_4_ after cross-evaluation, i.e., below the Global Atmosphere Watch (GAW) Programme of the World Meteorological Organization (WMO) compatibility goals (0.1 ppm for CO_2_ and 2 ppb for CH_4_^63^). The final merged datasets are referenced to the corresponding NOAA calibration scales, i.e., the WMO-X2019 CO_2_ scale^57^, the WMO-X2004A CH_4_ standard scale^34^, and the WMO-X2014A CO scale^35^.

**Fig. 5:**
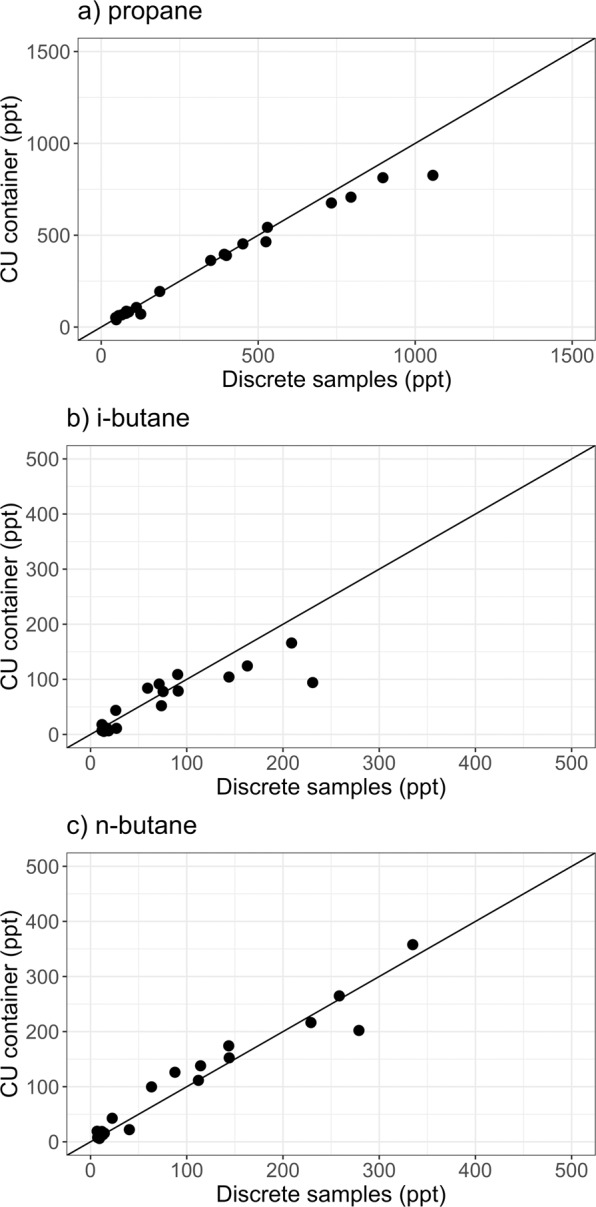
Comparison of redundant VOC measurements. Comparison of daily-averaged (**a**) propane, (**b**) i-butane, and (**c**) n-butane mole fractions measured in the University of Colorado (CU) container (y-axis) against atmospheric abundances inferred from discrete whole air sampling for post-cruise analysis at NOAA Global Monitoring Laboratory (x-axis). The black line is the bisector. Note that the CU VOC time series were not adjusted for calibration bias as we did not generate a merged VOC dataset.

Due to different sampling frequencies (3-hr vs. weekly snapshot samples), a direct cross-evaluation of redundant VOC measurements (propane, i-butane, n-butane) is not possible. Figure 5 shows the comparison of daily averages (n = 36) and highlights the very good agreement between the two datasets (correlation coefficients of 0.98, 0.85, and 0.93 for propane, i-butane, and n-butane, respectively; Spearman correlation test for paired samples). As no redundant measurements are available for DMS, SO_2_, and Hg(0), a similar cross-evaluation is not possible. Top-notch quality-control procedures were, however, used during the expedition (see Methods section) to ensure validity of the measurements.

## Usage Notes

The standardized *.txt file format permits easy import into all analysis software commonly used in the atmospheric science community. The files are self-explanatory as they contain all metadata and data. The time series archived on PANGAEA (Swiss container and merged datasets) and the Arctic Data Center (CU container and Met City datasets) are designed such that they can be used without further processing. The CO_2_, CH_4_, and CO merged datasets are referenced to the corresponding NOAA calibration scales, i.e., the WMO-X2019 CO_2_ scale^57^, the WMO-X2004A CH_4_ standard scale^34^, and the WMO-X2014A CO scale^35^. Most datasets contain a pollution flag indicating when local anthropogenic pollution was detected. Merged datasets do not include a pollution flag because they were created using clean individual time-series. For datasets collected in the CU container and at Met City, and for discrete flask sampling, no measurements were performed when the wind was out of the clean air sector (hence no pollution flag needed). When available, we highly encourage the use of hourly-averaged merged datasets that limit gaps in the time series. It should be noted that the time series available on the ARM user facility archive are not screened for local pollution nor adjusted for calibration bias. The raw chromatograms acquired with the automated GC-MS/FID system during the expedition are available in AIA format (*.CDF), one of the standard formats used for exchanging data among various chromatography systems.

## Data Availability

The pollution detection algorithm described in Beck *et al*.^30^ to identify and flag periods of primary polluted data in remote atmospheric time series is available at^64^: 10.5281/zenodo.5761101. The TERN peak fitting tool implemented in Igor Pro used for the analysis of the GC-MS/FID chromatograms is available at https://sites.google.com/site/terninigor/.
